# Characteristics and Outcomes of Children and Adolescents With Epithelial Ovarian Neoplasms From a Tertiary Cancer Centre in Western India: A Retrospective Study

**DOI:** 10.7759/cureus.84650

**Published:** 2025-05-22

**Authors:** Maharshi Trivedi, Pinky Meena, Himangi Tak, Dipesh Dave, Minu Chandra, Nitin Joshi, Harsha P Panchal, Jahnavi Gandhi

**Affiliations:** 1 Pediatric Oncology, The Gujarat Cancer and Research Institute, Ahmedabad, IND; 2 Pediatrics, All India Institute of Medical Sciences, Rajkot, IND; 3 Medical Oncology, The Gujarat Cancer and Research Institute, Ahmedabad, IND; 4 Pathology, The Gujarat Cancer and Research Institute, Ahmedabad, IND

**Keywords:** adolescent, children, india, outcome, ovarian epithelial carcinoma

## Abstract

Epithelial ovarian neoplasms (EON) are uncommon in children and data is limited. We conducted this study to assess the clinical characteristics and outcomes of children and adolescents with EON. Children ≤18 years of age diagnosed with EON, between 1st January 2010 and 31st December 2022 were included for retrospective analysis. Clinical characteristics, treatment details, and outcomes were noted. One-hundred-sixteen patients were diagnosed with ovarian mass, and eight (0.07%) of them were EON. Median age was 17 years (range: 13-18 years). One (12.5%) patient had a family history of breast cancer. One (12.5%) had borderline serous cystadenocarcinoma, four (50%) had malignant serous, and three (37.5%) had malignant mucinous adenocarcinoma. One girl with borderline disease (stage Ia) was treated with surgery and is alive. Among seven patients with malignant EONs, two (28.6%), two (28.6%), two (28.6%), and one (14.2%) had stage Ia, II, IIIc, and Iva disease, respectively. Two (28.6%) patients underwent primary debulking surgery. Two patients (28.6%) received neoadjuvant chemotherapy (NACT) followed by interval debulking. Three (42.8%) patients received chemotherapy and did not undergo surgery because of stable/progressive disease. Two patients (25%) with stage Ia were alive without evidence of disease. One girl (12.5%) with stage IIb relapsed after primary treatment and succumbed to the disease. All three patients with stage IIIc and one with stage IVa had relapse/progression, and three patients succumbed to the disease. Malignant EONs require multimodality treatment. Advanced disease and serous tumors are associated with poor survival. With a mean (± standard deviation) follow-up of 56 ± 10 months, the three-year progression-free survival (PFS) and overall survival (OS) were 37.5%±17.1% and 70%±18.2%, respectively.

## Introduction

Ovarian malignancies account for less than 1% of all pediatric and adolescent tumors [1]. Among them, 60% are germ cell tumors and less than 20% of them arise from surface epithelium. Almost half of surface epithelial tumors in children and adolescents are benign, and the rest are borderline ovarian tumors. Malignant epithelial ovarian neoplasms (EON) are exceptionally rare in the pediatric age group [1,2]. The histological profile of malignant EON is different from that observed in the adult population. Adolescent girls and young women with ovarian cancer are more likely to present with early-stage disease and low-grade tumors [1]. Management may differ in terms that these patients may desire future fertility and hormone function preservation [3,4]. In a population-based study, young women had a significant survival advantage across all stages. A five-year survival rate of 56% has been observed for women with stage III and IV tumors aged <30 years compared with only 22% for patients aged ≥60 years [5].

The data published on childhood EON are scarce and sporadic, primarily limited to case series [4,6], and include both benign and borderline EONs treated with heterogeneous treatment protocols [2,7,8]. Data on the unique clinicopathological characteristics, histological subtypes, treatment, and outcomes of malignant EON in children and adolescents are limited, especially for those treated with uniform protocols in lower-middle-income countries. We conducted this study to evaluate the outcomes of children and adolescents with EON treated at a tertiary care center using a uniform treatment protocol. The primary objective was to document disease progression and overall survival (OS). Secondary objectives included documenting the demographic and clinical characteristics, as well as treatment compliance, of these patients.

## Materials and methods

This retrospective review was conducted at the Pediatric Oncology unit, The Gujarat Cancer and Research Institute, Ahmedabad, Gujarat, India. Data of children aged 18 years or younger, pathologically diagnosed with epithelial ovarian cancer between January 1, 2010, and December 31, 2021, were retrieved from the hospital database system and analyzed retrospectively. Patients presenting with relapsed/refractory disease or a genetic predisposition syndrome were excluded. Permission to conduct the study was obtained from the Institutional Review Committee prior to initiation. Patients with benign ovarian tumors, germ cell tumors, sarcomas, and lymphomas were also excluded. Cancer antigen (CA125; ELISA assay), carcinoembryonic antigen (CEA; immunofluorescence assay), and CA 19-9 levels (ELISA assay), along with other blood investigations, were performed. Contrast-enhanced computed tomography (CT) scans of the chest, abdomen, and pelvis were used for staging. Each patient was staged according to the International Federation of Gynecology and Obstetrics (FIGO) 2014 classification system [9]. Data regarding clinical presentation, histology, stage of disease, treatment details, and outcomes were recorded. All patients were initially assessed for primary debulking surgery based on cross-sectional imaging. If surgery was deemed unfeasible, neoadjuvant chemotherapy (NACT) was initiated following a tru-cut biopsy. The feasibility of interval debulking surgery was reassessed after three cycles. If surgery remained unfeasible, chemotherapy was continued for a total of six cycles in the absence of progressive disease. In cases of progressive disease or if surgery remained unfeasible after six cycles, second-line salvage chemotherapy was considered. All children received paclitaxel and carboplatin as first-line chemotherapy, except for one girl who was treated with cyclophosphamide and cisplatin.

Statistical analysis

Continuous data were recorded as mean ± standard deviation (SD) and median ± interquartile range (IQR). Categorical data were recorded as frequency and percentage. For event-free survival (EFS) analysis, progression of disease, treatment abandonment, or death was considered an event. For progression-free survival (PFS), the progression of the disease was considered an event. Overall survival (OS) was defined as the time from diagnosis to death from any cause or last follow-up. Statistical analysis was performed using SPSS version 20. The Kaplan-Meier method was used to calculate survival.

## Results

A total of 116 patients were diagnosed with ovarian masses (both benign and malignant), of whom eight (0.07%) were diagnosed with EONs. The median age at diagnosis was 17 years (range: 13-18 years). One patient (12.5%) had a family history of breast cancer in her grandmother, three had no family history of cancer, and for the remaining patients, family history was not available. All patients presented with abdominal pain, and four (50%) also reported abdominal distension. All except one girl had attained menarche at the time of diagnosis.

Histopathological analysis revealed that one patient (12.5%) had borderline serous cystadenocarcinoma, four (50%) had malignant serous adenocarcinoma, and three (37.5%) had malignant mucinous cystadenocarcinoma. There were no cases of small cell, endometrioid, or transitional (Brenner) cell carcinoma. Among the seven patients with malignant ovarian carcinoma, four (57%) had low-grade tumors and one had a high-grade tumor (Table 1). CA-125 levels were elevated in six (75%) patients, all with serous histology (range: 201-2195 IU/dL), and in one patient with mucinous adenocarcinoma (42.88 IU/dL). CEA levels ranged from 0.27 to 4.73 IU/dL and were within normal limits in all patients. CA 19-9 levels were available for six patients; one had elevated levels (>37 U/L), who had borderline serous carcinoma.

**Table 1:** Characteristics and outcomes of patients with ovarian epithelial neoplasms HPE, histopathology examination; CA-125, cancer antigen-125; Lat, laterality; U, unilateral; B, bilateral; PDS, primary debulking surgery, IDS, interval debulking surgery; Lipo dox, liposomal doxorubicin

Sr. no.	Age (years)	HPE	CA-125 levels (U/mL)	Grade	Lat	Stage	Neoadjuvant chemotherapy	Surgery	Adjuvant chemotherapy	Event/outcome	Survival (months)	Treatment at relapse/progression	Outcome relapse/progression
1	16	Borderline serous	970		U	Ia	Not indicated	Optimal PDS	Not indicated	No disease/alive	79	-	-
2	17	Mucinous	23.5	1	U	Ia	Nil	Optimal PDS	Paclitaxel, carboplatin x 6	No disease/alive	57	-	-
3	17	Mucinous	25.9	1	U	Ia	Cisplatin, cyclophosphamide x 3	Not done	Nil	Abandoned/not reported back	7	-	-
4	17	Serous	412	1	U	IIa	Paclitaxel, carboplatin x 3	Not operable	NA	Progression/died	30	Lipo dox × 3 followed by surgery, followed by adjuvant chemotherapy	Died
5	13	Mucinous	42.8	1	U	IIb	Nil	Optimal PDS	Refused	Abandoned/presented with relapse/died	80	Paclitaxelcarboplatin x 3	Died
6	17	Serous	201	1	B	IIIc	Paclitaxel, carboplatin x 3	Suboptimal IDS	Paclitaxel, carboplatin x 3	Progression/alive	11	Lipo dox + bevacizumab x 6	On treatment/alive
7	18	Serous	4252	III	B	IIIc	Paclitaxel, carboplatin x 6	Suboptimal IDS	Nil	Abandoned post-3 cycle/progression after 6 cycles and surgery/died	49	Oral etoposide	Died
8	15	Serous	2195	1	U	IV	Paclitaxel, carboplatin x 4	Not done	NA	Progression/died	3	-	

Table 1 summarizes the characteristics and outcomes of all patients with EONs. One girl with borderline serous cystadenocarcinoma had stage Ia disease (Table 1, Patient 1). She was treated with fertility-preserving surgery (right salpingo-oophorectomy), which was optimal. She did not receive any adjuvant chemotherapy and is alive at 79 months post-surgery.

Among the seven patients with malignant EONs, two (28.6%) had stage Ia disease, two (28.6%) had stage II disease (one stage IIa and one stage IIb), two (28.6%) had stage IIIc disease, and one (14.2%) had stage IV disease at presentation.

Both patients with stage Ia disease had mucinous adenocarcinoma. One underwent a right oophorectomy before presenting to our center (Table 1, Patient 2), received six cycles of paclitaxel and carboplatin chemotherapy, and is alive without evidence of disease at 57 months of follow-up. The other (Patient 3) was started on NACT with cyclophosphamide and cisplatin but abandoned treatment after receiving three cycles.

One girl with stage IIa disease had serous papillary adenocarcinoma (Table 1, Patient 4). She was started on NACT, but surgery was not feasible after three cycles due to disease progression. She was then treated with second-line chemotherapy using liposomal doxorubicin, underwent surgery, and received adjuvant chemotherapy. She relapsed 30 months after the initial diagnosis and died from the disease. Another patient with stage IIb disease (Table 1, Patient 5) underwent optimal primary debulking surgery. However, her parents declined adjuvant chemotherapy. She relapsed with peritoneal, pleural, and lung metastases at 79 months and died due to disease.

Both patients with stage IIIc disease had serous adenocarcinoma. One (Table 1, Patient 6) underwent suboptimal interval debulking surgery after three cycles of NACT and received three cycles of adjuvant chemotherapy. She experienced disease progression at the end of treatment and was started on salvage chemotherapy with liposomal doxorubicin and bevacizumab. She showed a partial response and is currently awaiting surgery after six cycles. The other patient (Table 1, Patient 7), who had bilateral disease, started NACT but abandoned treatment after three cycles. She returned with disease progression three months later, received three additional cycles, and underwent suboptimal debulking surgery. She experienced disease progression 11 months after diagnosis and was placed on oral metronomic chemotherapy with palliative intent. She died 49 months after diagnosis.

The patient with stage IV disease (Table 1, Patient 8) presented with pleural effusion and pleural nodules. She was started on NACT and died after receiving four cycles of chemotherapy.

At a mean (±SD) follow-up of 56 (±10) months, the three-year PFS and OS rates were 37.5% (±17.1%) and 70% (±18.2%), respectively. Among the seven patients with malignant EONs, three are alive (two without evidence of disease and one with active disease), one is lost to follow-up, and four have died due to the disease. Three patients abandoned or refused the prescribed treatment: one never returned for follow-up, and two relapsed or progressed and subsequently died. When treatment abandonment was considered an event, the three-year EFS was 25% (±15.3%).

Three children showed disease progression during or at the end of treatment (all platinum-refractory). Two died of the disease, and one is currently alive with the disease on salvage chemotherapy.

## Discussion

Here, we present a retrospective review of children and adolescents diagnosed with EON over an 11-year period at a tertiary cancer center. The majority of the children had low-grade (grade 1) malignant histology. We observed that serous carcinoma was the most common histologic subtype among children presenting with malignant EON. These patients tended to present with advanced-stage disease (more than one-third of serous carcinoma cases in our study) and had worse outcomes compared to those with mucinous tumors. One-third of the evaluated children did not receive the prescribed treatment due to abandonment and poor compliance. Children with malignant EONs, especially those presenting with advanced-stage disease, had poor outcomes.

Ovarian malignancies are rare in children and adolescents, accounting for less than 1% of all malignant tumors [6]. Epithelial ovarian tumors constitute 10%-20% of all ovarian tumors in this age group [6,10]. They usually occur after menarche, primarily in adolescents older than 15 years [4,11]. Proposed hypotheses for the development of epithelial ovarian tumors include: a) hormonal stimulation of the ovary, and b) repeated disruption and repair of the ovarian surface epithelium during ovulation, which predisposes to a higher risk of spontaneous mutation [2]. These tumors commonly present with acute or chronic abdominal pain and abdominal distension. Occasionally, they present as a surgical emergency due to ovarian torsion and are diagnosed intraoperatively [2]. In our series, most patients were late adolescents (median age 17 years) at presentation; only one child had not attained menarche at diagnosis. All patients presented with classic symptoms of abdominal pain and distension. None presented with ovarian torsion.

In children and adolescents, benign cystadenomas constitute the majority of EONs, followed by borderline ovarian tumors. Various series report that benign and borderline tumors range from 30% to 100% of reported EON cases in this population [2,8,10]. Malignant epithelial tumors are relatively uncommon in children and adolescents [2,10]. This contrasts with our observation, where 87.5% of tumors were malignant. This discrepancy may be due to referral bias, as our tertiary cancer center typically manages more complex cases, while many benign and borderline tumors are treated at peripheral and secondary care centers without referral. Histopathologically, serous and mucinous tumors are the most common types in children and adolescents, whereas clear cell, endometrioid, and transitional (Brenner) histologies are exceedingly rare [1,2]. In our series, all tumors were either serous or mucinous. Serous tumors were more common and more likely to present with advanced disease, bilateral involvement, and higher grade compared to mucinous tumors [1,4]. Both children with bilateral disease and all children with advanced-stage (III and IV) tumors had serous histology in our study. However, pediatric malignant epithelial tumors demonstrate a strikingly different histologic profile compared to adults. While high-grade serous carcinomas are the most common malignant ovarian tumors in adults, their low-grade counterparts are rare, accounting for only 10% of such tumors. In children, most malignant epithelial ovarian tumors are low-grade [1,4]. Similarly, in our study, 85% of malignant EONs were low-grade (grade 1).

Surgery remains the cornerstone of treatment for benign and borderline EONs. The conventional surgical approach is salpingo-oophorectomy with removal of all visible omental and peritoneal implants, without hysterectomy or contralateral oophorectomy [2,4,10,12-14]. Several authors report that adjuvant therapy is generally not required post-surgery, even in stage III disease, for benign and borderline EONs [2,13]. Using this approach, survival rates for benign and borderline EONs approach 97% to 100% in most reports [1,2,4,8,10,13]. In our series, one patient with a borderline neoplasm underwent primary debulking surgery without adjuvant treatment and remains disease-free at 79 months follow-up. Since these patients can experience late relapse, long-term follow-up is necessary [1,15]. Routine follow-up includes imaging (ultrasound, magnetic resonance imaging) and blood marker sampling (CA-125). Normalization of CA-125 after the first cycle of chemotherapy is associated with improved survival in early-stage ovarian cancer.

Malignant EONs in children and adolescents are treated using similar protocols and principles as adults. Low-risk early-stage EONs (stage Ia, Ib, grade I tumors) are treated with surgery alone [1,14,16]. High-risk early-stage EONs (stage Ia, Ib grade II-III; stage Ic-II of all grades) should receive three to six cycles of cisplatin- or carboplatin-based chemotherapy post-surgery, with carboplatin and paclitaxel being the most commonly used regimen [17-19]. For stage III and IV disease, six to eight cycles of chemotherapy are recommended. Surgery may be performed upfront or following NACT [20]. Some protocols also incorporate bevacizumab as part of chemotherapy and for post-chemotherapy maintenance [21,22]. For adult patients with germline or somatic BRCA1 or BRCA2 mutations, poly ADP-ribose polymerase (PARP) inhibitors are recommended as maintenance therapy [23,24]. PARP inhibitors are currently under trial in pediatric patients [25]. Surgery is performed via open laparotomy and typically includes hysterectomy, bilateral salpingo-oophorectomy, omentectomy, and debulking of peritoneal implants (often including bowel or adjacent organ resection if necessary) to achieve microscopic residual disease [26]. Some reports suggest that salpingo-oophorectomy without lymph node dissection, omentectomy, or hysterectomy can be performed in stage I malignant EONs without increasing relapse risk [4]. In our series, patients with malignant EON were treated with surgery and chemotherapy following adult treatment recommendations.

Survival rates for malignant EON range from 50% to 80% in different reports [1,4]. Early-stage malignant tumors have survival rates exceeding 90%, while advanced-stage EON survival is around 50% [4]. Pediatric survival rates are comparable to those in adults with low-grade serous carcinoma, suggesting that OS, natural history, and chemotherapy response in pediatric malignant surface epithelial neoplasms mirror those seen in adults [1]. In our study, only two children were alive at the last follow-up. However, over one-third of patients did not receive prescribed treatment due to abandonment and poor compliance, most of whom had early-stage disease. Ensuring treatment compliance in these patients could have improved survival. Among patients who completed planned treatment, 60% had advanced-stage disease, and 50% were alive at the last follow-up. Serous malignant tumors had worse outcomes compared to mucinous tumors [1,4]. In our series, 66% of patients with mucinous tumors were alive, compared to only 25% of those with serous histology.

The strength of our study lies in its evaluation of children and adolescents with EON treated over 11 years using a uniform treatment protocol at a tertiary cancer center, with long-term follow-up. There are very few reports on the epidemiology and characteristics of EONs from low- and middle-income countries, and survival data for children with EONs from these regions is scarce. Limitations of our study include its retrospective design, limited patient numbers, and the major impact of poor compliance and treatment abandonment on survival outcomes. Additionally, referral bias may exist, as benign and borderline cases may have been managed at primary or secondary healthcare centers without referral to our center.

## Conclusions

Benign and borderline EONs can be cured with surgery alone and are associated with excellent outcomes. Malignant EONs require multimodal treatment, following protocols similar to those used in adults. Children with advanced-stage disease and serous histology have poorer survival. Uniform treatment protocols should be rigorously implemented in children with surface epithelial ovarian neoplasms. Genetic testing and counseling are essential components of the management of children with EON.
